# Utility of Translocator Protein (18 kDa) as a Molecular Imaging Biomarker to Monitor the Progression of Liver Fibrosis

**DOI:** 10.1038/srep17327

**Published:** 2015-11-27

**Authors:** Akiko Hatori, Joji Yui, Lin Xie, Katsushi Kumata, Tomoteru Yamasaki, Masayuki Fujinaga, Hidekatsu Wakizaka, Masanao Ogawa, Nobuki Nengaki, Kazunori Kawamura, Feng Wang, Ming-Rong Zhang

**Affiliations:** 1 Molecular Imaging Center, National Institute of Radiological Sciences, 4-9-1 Anagawa, Inage-ku, Chiba 263-8555, Japan; 2 Department of Nuclear Medicine, Nanjing First Hospital, Affiliated to Nanjing Medical University, 68 Chanle Road, Nanjing 210006, China

## Abstract

Hepatic fibrosis is the wound healing response to chronic hepatic injury caused by various factors. In this study, we aimed to evaluate the utility of translocator protein (18 kDa) (TSPO) as a molecular imaging biomarker for monitoring the progression of hepatic fibrosis to cirrhosis. Model rats were induced by carbon tetrachloride (CCl_4_), and liver fibrosis was assessed. Positron emission tomography (PET) with *N*-benzyl-*N*-methyl-2-[7,8-dihydro-7-(2-[^18^F]fluoroethyl)-8-oxo-2-phenyl-9*H*-purin-9-yl]-acetamide ([^18^F]FEDAC), a radioprobe specific for TSPO, was used for noninvasive visualisation *in vivo*. PET scanning, immunohistochemical staining, *ex vivo* autoradiography, and quantitative reverse-transcription polymerase chain reaction were performed to elucidate the relationships among radioactivity uptake, TSPO levels, and cellular sources enriching TSPO expression in damaged livers. PET showed that uptake of radioactivity in livers increased significantly after 2, 4, 6, and 8 weeks of CCl_4_ treatment. Immunohistochemistry demonstrated that TSPO was mainly expressed in macrophages and hepatic stellate cells (HSCs). TSPO-expressing macrophages and HSCs increased with the progression of liver fibrosis. Interestingly, the distribution of radioactivity from [^18^F]FEDAC was well correlated with TSPO expression, and *TSPO* mRNA levels increased with the severity of liver damage. TSPO was a useful molecular imaging biomarker and could be used to track the progression of hepatic fibrosis to cirrhosis with PET.

Hepatic fibrosis is the response to chronic hepatic injury resulting from various factors, such as alcohol abuse, viral infection, or cholestasis, and is characterised by the excessive production and deposition of extracellular matrix (ECM) due to loss of liver cells during hepatitis. Importantly, hepatic fibrosis can progress to cirrhosis^1^,^2^. In Japan, cirrhosis is mainly caused by hepatitis virus, with hepatitis C virus accounting for 70% of cases and hepatitis B virus accounting for 20% of cases, as reported by the National Center for Global Health and Medicine (Chiba, Japan). Persistent infection in hepatitis virus results in progression to chronic hepatitis, cirrhosis, and hepatocellular carcinoma by 20–30 years after infection, without a history of noticeable symptoms reported by patients. Therefore, development of sensitive diagnostic methods to visualise and monitor hepatic fibrosis is needed. This information on the presence, degree and progression of liver fibrosis is useful for making therapeutic decisions or predicting disease outcomes^3^.

In recent years, various evaluation methods for hepatic fibrosis have been developed, generally involving the use of serum markers and the measurement of liver stiffness (i.e., using transient elastography and magnetic resonance elastography)^3^,^4^,^5^,^6^. Positron emission tomography (PET) imaging with [^18^F]fluorodeoxyglucose is useful for direct, quantitative, and multispatial visualisation of physiological and cellular processes of hepatocellular carcinoma at multiple time points and at the macroscopic level^7^. However, no studies have reported the use of PET imaging for noninvasive diagnosis of hepatic fibrosis.

Inflammation is a common feature observed during hepatic fibrogenesis in chronic liver diseases^8^. As the inflammatory response progresses, the expression levels of translocator protein (18 kDa) (TSPO), a receptor complex primarily expressed in mitochondria, are increased in inflammatory cells^9^,^10^. TSPO has a variety of biological functions, such as cholesterol transport, steroid hormone synthesis, apoptosis, cell proliferation, porphyrin and heme transport, anion transport, mitochondrial function, immunomodulation, and inflammation^10^. We have identified TSPO as a potential imaging biomarker for visualising various inflammatory diseases in living subjects. For example, PET studies with *N*-benzyl-*N*-methyl-2-[7,8-dihydro-7-(2-[^18^F]fluoroethyl)-8-oxo-2-phenyl-9*H*-purin-9-yl]-acetamide ([^18^F]FEDAC), a TSPO-specific radioprobe, have been carried out to noninvasively visualise neuroinflammation^11^, lung inflammation^12^, acute liver damage^13^, and nonalcoholic fatty liver disease^14^. Moreover, transformed hepatic stellate cells (HSCs), the major ECM-producing cells during liver fibrosis, have been shown to express TSPO both *in vitro* and *in vivo*^15^. Thus, TSPO may represent an effective biomarker for monitoring the progression of liver fibrosis.

In this study, we aimed to evaluate potential utility of TSPO as a molecular imaging biomarker for noninvasive monitoring of the progression of hepatic fibrosis to cirrhosis. To this end, we used a rat model of cirrhosis in which hepatic fibrosis was induced by chronic carbon tetrachloride (CCl_4_) injection^16^,^17^,^18^,^19^. We then measured the uptake of [^18^F]FEDAC in livers using a PET scanner at various times after CCl_4_ treatment and evaluated the progression of hepatic fibrosis and the expression of TSPO by histological observation. Our data provide important insights regarding the utility of TSPO as a marker of hepatic fibrosis.

## Results

### Histological analysis

**Figure 1:**
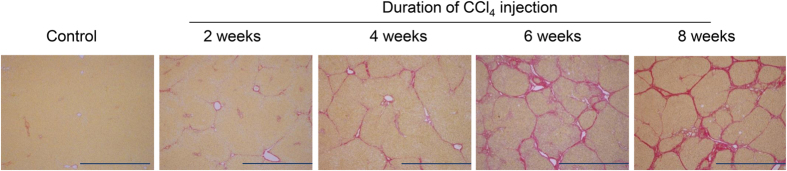
Progression from fibrosis to cirrhosis in rat livers induced by chronic CCl_4_ treatment. Sirius red-stained liver sections from control rats and rats treated with CCl_4_ for 2, 4, 6, or 8 weeks, showing progression from liver fibrosis to cirrhosis. Scale bar: 1 mm.

Figure 1 shows representative Sirius red-stained images reflecting histological alterations in liver sections from control rats and rats treated with CCl_4_ for 2, 4, 6, or 8 weeks. Table 1 shows the fibrosis scores and percentages of fibrotic regions in livers. Livers of CCl_4_ treatment groups exhibited gradual hepatic parenchymal alterations in centrilobular areas, increasing with the duration of treatment. After 2 weeks of treatment, fibrous central venule to central venule (C-C) bridging septa with deposition of thin collagen fibres were observed, and the fibrotic region was increased to 2.68% ± 0.16% of the liver sections compared to 0.36% ± 0.08% of that in the control group. After 4 weeks of treatment, multiple C-C fibrotic septa were incompletely connected and centred by the portal tract. The percentage of the fibrotic region was increased to 5.82% ± 0.84%. After 6 weeks of treatment, C-C fibrotic septa were completely connected, and moderate central venule to portal tract (C-P) bridging septa divided the pseudolobule. After 8 weeks of treatment, the number of smaller-sized nodules was increased, and 3 of 5 rats showed complete cirrhosis. The fibrotic regions of livers from rats treated for 6 and 8 weeks were further increased to 12.45% ± 1.16% and 13.79% ± 1.99%, respectively.

### PET study

**Figure 2:**
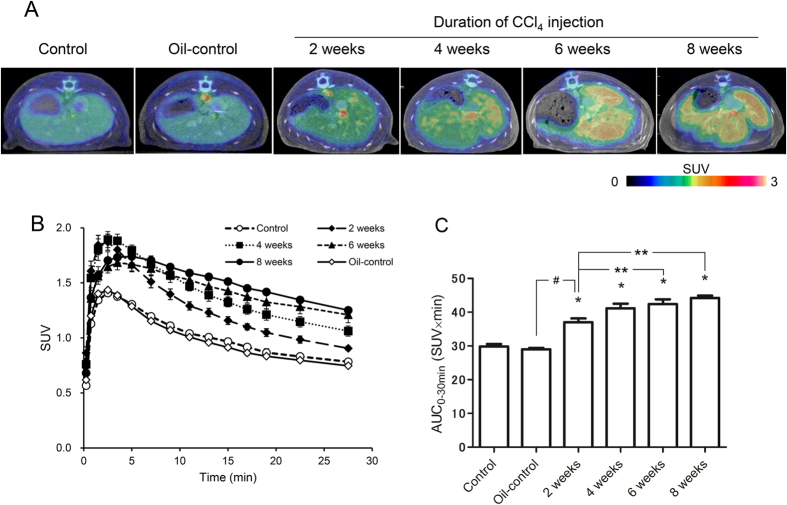
Accumulation of radioactivity in the livers of control and CCl_4_-treated rats. (**A**) Representative transverse PET/CT fusion images of rat livers. PET images were acquired between 0 and 30 min after injection of [^18^F]FEDAC. The pseudocolour bar represents the radioactivity level in the liver. (**B**) Time-activity curves in livers (n = 4 for each group). (**C**) Radioactivity values (AUC_0–30 min_, SUV × min, n = 4, mean ± SE) calculated from the time-activity curves between 0 and 30 min. *p < 0.05 for control versus CCl_4_ treatment for 2, 4, 6, or 8 weeks. **p < 0.05 for 2 weeks CCl_4_ treatment versus CCl_4_ treatment for 6, or 8 weeks. ^#^*p* < 0.05 for 2 weeks oil control versus 2 weeks CCl_4_ treatment.

To visualise liver damage in CCl_4_-treated rats, PET scans were performed for 30 min after injection of [^18^F]FEDAC. Figure 2A shows representative PET summation images between 0 and 30 min. PET imaging in the two-week oil-control group was similar to that in control group. Radioactivity in the livers increased with the increasing duration of CCl_4_ treatment as compared with that in control livers. Additionally, the liver shape became irregular after 6 and 8 weeks CCl_4_ treatment, demonstrating the disorganisation of the lobular architecture.

Figure 2B shows time-activity curves which radioactivity is expressed as the standardised uptake value (SUV). Figure 2C shows uptake values, represented as area under the time activity curves (AUC_0–30 min_), in the livers. The accumulation of radioactivity in the livers increased significantly in all treatment groups as compared to that in the control and oil-control groups (p < 0.0001). Specifically, while radioactivity levels in the control group were 29.9 ± 0.7, those in livers of rats treated with CCl_4_ for 2, 4, 6, and 8 weeks were 37.0 ± 1.1 (1.2-fold), 41.2 ± 1.4 (1.4-fold), 42.4 ± 1.4 (1.4-fold), and 44.2 ± 0.7 (1.5-fold), respectively. In the oil-control group, radioactivity levels were 29.0 ± 0.3.

### Immunohistochemical staining results

**Figure 3:**
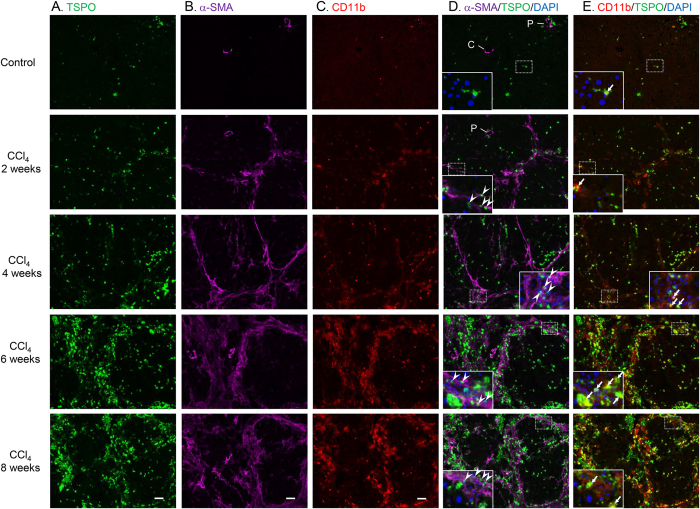
Increased levels of TSPO expressed in HSCs and macrophages from liver tissues after CCl_4_ treatment. Triple immunofluorescence labelling of TSPO (**A**., green), α-SMA (**B**., pink), CD11b (**C**., red), and DAPI (blue) in liver sections from control animals and animals treated with CCl_4_ for 2, 4, 6, or 8 weeks (**A–E**). (**D,E**) show merged images of (**A,B**) and of (**A,C**), respectively. The dotted line square in each slide (**D,E**) is enlarged and shown in the inset with DAPI. Arrowheads point to TSPO double-stained HSCs (α-SMA-positive cells). Arrows point to TSPO double-stained macrophages (CD11b-positive cells). Abbreviations: **C**, central venule; P, portal tract. Scale bar: 50 μm.

Triple immunofluorescence staining of TSPO (green), α-smooth muscle actin (α-SMA) (pink), CD11b (red), and DAPI (blue) in control and CCl_4_-treated livers is shown in Fig. 3A–E. In the controls, hepatic macrophages (CD11b-positive cells) expressing TSPO, mainly comprised of Kupffer cells, were observed in some cells. α-SMA-stained blood vessels were observed, but no cells exhibited double staining for α-SMA and TSPO in control livers. Additionally, TSPO expression was low in hepatocytes, while intense fluorescence signals were observed in the hepatic arteriole and intralobular bile duct of the portal tract in all experimental groups, including the control.

After 2 weeks of treatment, activated HSCs (α-SMA-positive cells) were observed in the pericentral zone and fibrotic septa. TSPO double-stained HSCs were also found in these areas. Macrophages expressing TSPO were located not only in hepatic sinusoids, but also adjacent to fibrotic septa. After 4 weeks of treatment, hepatic fibrosis progressed, and fibrotic septa became thick. The number of HSCs expressing TSPO was significantly increased around the fibrotic septa and the periportal area, and these cells also infiltrated the lobules from the edges of the septa. The number of macrophages expressing TSPO was also increased in hepatic sinusoids and nearby activated HSCs. After 6 weeks of treatment, liver fibrosis developed progressively to become thick fibrotic bands, and the pseudolobule was divided into smaller nodules by C-P bridging septa. The numbers of HSCs and macrophages expressing TSPO increased progressively; these cells localised around the rampant fibrotic area and inside the nodules. Finally, after 8 weeks of treatment, the number of smaller-sized nodules was increased, indicating the development of cirrhosis. The locations of HSCs and macrophages expressing TSPO were similar to those in the 6-week treatment group.

### Autoradiography and TSPO expression

**Figure 4:**
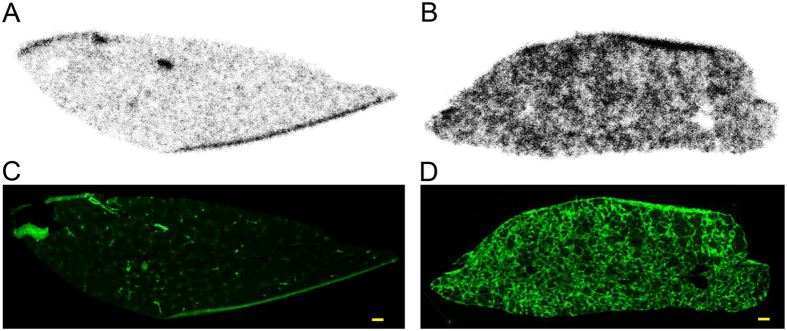
Distributions of radioactivity and TSPO expression in control and CCl_4_-treated rat livers. Autoradiographic images of liver sections 1 h after injection of [^18^F]FEDAC are shown in (**A**) and (**B**) for control rats and rats treated with CCl_4_ for 6 weeks, respectively. Immunofluorescence staining of TSPO using the same sections as in (**A**) and (**B**) are shown in (**C**) and (**D**) for control rats and rats treated with CCl_4_ for 6 weeks, respectively. The distribution of radioactivity from [^18^F]FEDAC correlated well with the netlike appearance of TSPO expression in the hepatic lobules (**B,D**). Scale bar: 1 mm.

The distribution of radioactivity in hepatic lobules at 1 h after injection of [^18^F]FEDAC was compared between control rats and rats treated with CCl_4_ for 6 weeks using autoradiography (Fig. 4A,B). Additionally, TSPO distribution by immunofluorescence staining is shown in Fig. 4C,D. After 6 weeks of treatment, [^18^F]FEDAC levels correlated well with the netlike distribution of TSPO expression in hepatic lobules. In section from the control group, radioactivity and TSPO levels were low. The edges of sections occasionally showed artificially higher signals due to the folding and thickening of the sections.

### Gene expression associated with TSPO

**Figure 5:**
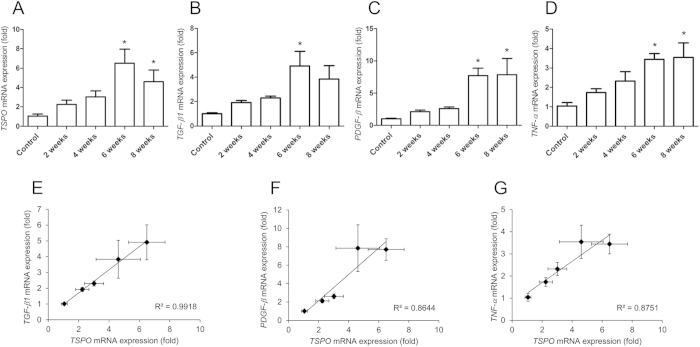
Effects of CCl_4_ treatment on the expression of *TSPO* mRNA. Expression of hepatic *TSPO* (**A**), *TGF-β1* (**B**), *PDGF-β* (**C**), and *TNF-α* (**D**) mRNA in control rats and rats treated with CCl_4_ for 2, 4, 6, or 8 weeks. *p < 0.05 for the control versus CCl_4_ treatment for 6 or 8 weeks. Correlations between *TSPO* mRNA expression levels and *TGF-*β*1* (**E**), *PDGF-*β (**F**), and *TNF-*α (**G**) mRNA expression levels.

Finally, to provide insights into the mechanisms involved in hepatic fibrosis associated with TSPO function, we analysed the expression levels of *TSPO, transforming growth factor (TGF)-*β*1, platelet-derived growth factor (PDGF)-*β, and *tumour necrosis factor (TNF)-*α mRNAs by quantitative reverse-transcription polymerase chain reaction (qRT-PCR) (Fig. 5A–D). Hepatic *TSPO* mRNA expression was increased after 2 (2.2-fold), 4 (3.0-fold), 6 (6.5-fold), and 8 weeks (4.6-fold) of CCl_4_ treatment as compared with that in the control group. Hepatic *TGF-*β*1, PDGF-*β, and *TNF-*α mRNA levels were also increased by 4.9-, 7.6-, and 3.3-fold, respectively, after 6 weeks of CCl_4_ treatment, as compared with those in the control. Importantly, the expression of *TSPO* mRNA increased with the increase in severity of liver damage. Furthermore, increased *TSPO* mRNA expression correlated with the increases in *TGF-*β*1, PDGF-*β, and *TNF-*α mRNAs (Fig. 5E–G).

## Discussion

Liver biopsy, the gold standard for accurate assessment of fibrosis, is associated with the potential for complications, patient discomfort, and poor reproducibility due to sampling errors; therefore, more advanced approaches are required to provide complementary information for diagnoses and monitoring^3^,^6^. Indeed, several evaluation methods for hepatic fibrosis by using serum markers and imaging modalities, such as transient elastography and magnetic resonance elastography, have been developed in recent years^3^,^4^,^5^,^6^. Transient elastography is widely used in Europe to measure liver stiffness; however, this technology is not sensitive enough to enable accurate assessment of fibrosis progression over time in individual patients^4^,^6^. To this end, in the present study, we aimed to examine whether TSPO could be used as a molecular imaging biomarker for the progression of hepatic fibrosis. Our results demonstrated that PET imaging with the TSPO-specific radioprobe [^18^F]FEDAC permitted noninvasive visualisation of the progression from hepatic fibrosis to cirrhosis. Thus, this method may have important clinical uses.

In this study, our experimental model involved induction of hepatic fibrosis by treatment with CCl_4_. This model has been extensively applied in many previous studies^16^,^17^,^18^,^19^. CCl_4_ is activated by cytochrome P450 to form trichloromethyl radical^20^, which attacks organelles in hepatocytes, causes necrosis of parenchymal cells, and promotes inflammatory responses in the liver^17^. Histological analysis revealed the gradual progression from hepatic fibrosis to cirrhosis with the increasing duration of CCl_4_ treatment. Moreover, the observed histological changes indicated that the primary cellular source of ECM in hepatic fibrosis was activated stellate cells. Fibrogenic cells are derived not only from resident stellate cells, but also from portal fibroblasts, circulating fibrocytes, bone marrow, and the epithelial-mesenchymal cell transition^21^. The relative contribution of each source is likely to depend on the varying etiologies of liver injury^21^.

In rats treated with CCl_4_ for up to 8 weeks, PET/CT images with [^18^F]FEDAC could track the progression of liver damage (Fig. 2). Comparisons of liver images and AUC_0–30 min_ values between the control and CCl_4_-treated groups indicated that the heterogeneous distribution of radioactivity and radioprobe binding were elevated as the duration of CCl_4_ treatment and the severity of liver damage increased. Moreover, after 6 and 8 weeks of CCl_4_ treatment, livers acquired an irregular shape and disorganised lobular architecture. Our results also revealed that the distribution of radioactivity from [^18^F]FEDAC was associated with TSPO binding in the fibrotic liver.

Interestingly, we found that few TSPO-positive Kupffer cells were present in control livers. However, after CCl_4_ treatment, activated HSCs were observed in the pericentral zone and fibrotic septa, and CD11b and TSPO double-positive macrophages were located in hepatic sinusoids and adjacent to fibrotic septa. Liver fibrosis developed progressively, and the numbers of TSPO-expressing HSCs and macrophages were increased progressively (Fig. 3). The interaction between Kupffer cells and HSCs has been reported to increase during development of liver fibrosis in CCl_4_-treated rats^22^, and activated HSCs and macrophages/Kupffer cells have been suggested to stimulate each other using secreted cytokines, promoting the development of liver fibrosis^23^. Consistent with this, macrophages and Kupffer cells are almost always found in close proximity to collagen-producing myofibroblasts^22^,^23^.

HSC activation is triggered by the release of mitogenic PDGF and epidermal growth factor from activated HSC and TGF-β1, mostly from Kupffer cells and macrophages^1^,^2^. Moreover, TGF-β1 promotes fibrosis by accelerating HSC differentiation into myofibroblasts, enhancing the expression of tissue inhibitors of matrix metalloproteinases that block ECM degradation, and directly promoting synthesis of interstitial fibrillar collagens^2^. Activated HSCs also release TGF-β^24^. In contrast, PDGF acts as a potent profibrotic signal by stimulating the proliferation of activated and collagen-producing HSCs^21^. TNF-α, which is primarily produced by Kupffer cells^22^,^25^, is a pro-inflammatory cytokine that has important functions in the pathogenesis of liver fibrosis. The mechanism and mediators by which macrophages promote HSC activation are not completely understood^26^. In this study, we found that the expression levels of hepatic *TSPO, TGF-*β*1, PDGF-*β, and *TNF-*α mRNAs were increased progressively by 6 weeks of CCl_4_ treatment (Fig. 5), at which time liver fibrosis had almost developed into cirrhosis. Positive correlations were detected between *TSPO* expression and the expression of *TGF-*β*1, PDGF-*β, and *TNF-*α. These results showed that TSPO gene expression increases in the liver during fibrogenesis in association with pro-fibrotic genes. TSPO mRNA increases are likely in HSCs and macrophages/Kupffer cells since those two cell types express more TSPO protein after chronic liver injury (Fig. 3); however, further gene expression studies in isolated pure cell populations are needed to confirm. Thus, these findings showing the upregulation of TSPO and related factors during liver fibrosis may be characteristic features of the pathophysiological abnormalities occurring during the progression of fibrosis. Although the pathogenic mechanism is different between the hepatitis and CCl_4_ models, TSPO may also be increased in the liver tissues of patients with hepatitis. It is noted that only using PET, it is difficult to distinguish which radioactive signal from the certain cellular source in the present model, and to distinguish between fibrosis and hepatitis without fibrosis. Therefore, definitive diagnosis of these liver diseases should be performed using other modalities, such as biopsy. Despite the limitations of PET modality for diagnosis, our present findings demonstrate that the progression of hepatic fibrosis in the CCl_4_ model may provide important insights into the potential role of PET/CT imaging for follow-up assessments of patients with hepatitis.

In summary, we demonstrated that TSPO was a useful imaging biomarker for noninvasive monitoring of liver fibrosis. The results at multiple time points indicated significant increases in TSPO expression corresponding with liver damage. Thus, PET imaging studies with the TSPO-specific radioprobe [^18^F]FEDAC may be useful for noninvasive visualisation of the progression from hepatic fibrosis to cirrhosis in patients with chronic liver diseases.

## Materials and Methods

### Production of [^18^F]FEDAC

[^18^F]FEDAC was prepared in-house by reaction of a precursor with [^18^F]fluoroethyl bromide, with more than 98% radiochemical purity and 140–210 GBq/μmol specific activity (n = 16)^11^.

### Study animals

Male Sprague-Dawley rats (6–7 weeks old) were purchased from Japan SLC (Shizuoka, Japan). All animals received humane care, and all experiments were approved by the Animal Ethics Committee of the National Institute of Radiological Sciences (permit number: 10-1005, Chiba, Japan). All experiments were carried out according to the recommendations of the Committee for the Care and Use of Laboratory Animals, National Institute of Radiological Sciences.

### Preparation of a rat model of liver fibrosis

A total of 36 healthy rats were used in this experiment after appropriate acclimation to the housing conditions. The rats were anaesthetised with 5% (v/v) isoflurane and injected intraperitoneally (i.p.) with CCl_4_ (2 mL/kg, dissolved 1:1 in sterile olive oil) twice per week. Small-animal PET studies were performed at 3 days after 2, 4, 6, and 8 weeks of CCl_4_ treatment, with four rats per time point. Two control groups consisted of four rats per each group were set for PET studies as follows; the control group with no treated rats and the oil-control group with 2 weeks of olive oil treated rats (1 mL/kg, 2 times/week, i.p.). For the control group, four normal rats were used without CCl_4_ treatment. At least four animals for each group were sacrificed at 3 days after 2, 4, 6, and 8 weeks of CCl_4_ treatment. A part of each rat liver was excised and fixed in 10% neutral buffered formalin for histopathological evaluation. The remaining liver samples were stored at −80 °C for qRT-PCR and immunohistochemical examination. *Ex vivo* autoradiography was performed using three rats treated with CCl_4_ for 6 weeks and three control rats.

### Histological analysis

Fixed liver samples were embedded in paraffin and cut into 4-μm-thick sections for staining with hematoxylin and eosin or Sirius red. Fibrosis was assessed histologically as follows: score 0, no fibrosis; score 1, small portion of fibrous tissue in the central venule (C); score 2, fibrous C-C septa appearance; score 3, multiple C-C fibrotic septa incompletely connected; score 4, C-C septa completely connected (pseudolobule), occasional C-P bridging appearance; score 5, moderate C-P bridging septa further dividing the pseudolobule, with a number of smaller-sized nodules comprising less than 50% (incomplete cirrhosis); score 6, multiple C-P bridging septa further dividing the pseudolobule, with a number of smaller-sized nodules comprising more than 50% (complete cirrhosis)^18^,^19^. Histologic evaluation was performed by two pathologists in a random, blinded manner.

The percentage of fibrosis was determined from 10–15 microscopic fields (magnification, × 40) of Sirius red-stained slides per animal for collagen quantification using Win ROOF software (Mitani, Tokyo, Japan) with a microscope (Olympus, Tokyo, Japan).

### Small-animal PET/CT scans

PET scans were performed using a small-animal Inveon PET scanner (Siemens, Knoxville, TN, USA), which provides 159 transaxial slices with 0.796-mm (centre-to-centre) spacing, a 10-cm transaxial field of view, and a 12.7-cm axial field of view. Before scanning, rats were anesthetised with 5% (v/v) isoflurane and maintained thereafter with 1–2% (v/v) isoflurane. Emission scans were acquired for 30 min in three-dimensional (3D) list mode with an energy window of 350–750 keV immediately after intravenous injection of [^18^F]FEDAC (10 MBq/0.16–0.25 mL, 40–80 pmol) into the rats (n = 4 for each group).

All list-mode acquisition data were sorted into 3D sinograms, which were then Fourier rebinned into two-dimensional (2D) sinograms (frames × min: 2 × 0.5, 3 × 1, 8 × 2, 2 × 5). Dynamic images were reconstructed with filtered back-projection using a Hanning filter and a Nyquist cutoff of 0.5 cycles/pixel. A region of interest was placed on the liver using ASIPro VM (Analysis Tools and System Setup/Diagnostics Tool; Siemens Medical Solutions USA). The uptake of radioactivity in the liver was decay-corrected to the injection time and expressed as SUV, which was normalised to the injected radioactivity and body weight. The SUV was calculated as follows: SUV = (radioactivity per millilitre tissue /injected radioactivity) × gram body weight. AUC_0–30 min_ (SUV × min) for liver was calculated from 0 to 30 min after injection.

After PET scans, rats remained under 1–2% (v/v) isoflurane. Computed tomography (CT) attenuation data were acquired on nonenhanced scans with the breath-holding model for 2.5 min using a small-animal CT system (R_mCT2, Rigaku, Tokyo). Images were acquired with the X-ray source set at 90 kVp and 200 μA. Average CT attenuation images and dynamic PET images were reconstructed and fused using IRW 4.0.

### Immunohistochemical staining assay

Frozen liver samples were cut in-house into 10-μm-thick slices using a cryotome (HM560, Carl Zeiss, Oberkochen, Germany) for immunohistochemical examinations. Fresh frozen sections were soaked in methanol containing 0.3% H_2_O_2_ for 30 min at room temperature to fix and block endogenous peroxidase activity, and then washed with PBS. Triple staining with primary antibodies was performed using a rabbit anti-mouse TSPO antibody (NP155, 1:1000)^27^ and a monoclonal mouse anti-rat CD11b antibody (1:100; AbD Serotec, Raleigh, NC, USA) for identifying Kupffer cells and macrophages in the liver. The sections were incubated with the primary antibodies overnight at 4 °C. After the first immunoreaction, the sections were incubated with fluorophore-conjugated (Alexa Fluor 546) goat anti-mouse IgG secondary antibody (1:500; Invitrogen, Camarillo, CA, USA) and biotin-conjugated goat anti-rabbit IgG secondary antibody (1:1000; Invitrogen) for 1 h at room temperature, followed by tyramide signal amplification using a Fluorescein System (FITC) (PerkinElmer, Waltham, MA, USA). After washing with PBS, the sections were incubated with monoclonal mouse anti- α-SMA antibody (1:400; Sigma-Aldrich, St. Louis, MO, USA) for 1 h at room temperature, followed by fluorophore-conjugated (Alexa Fluor 647) chicken anti-mouse IgG secondary antibody (1:500; Thermo Fisher, Rockford, IL, USA). The sections were washed with PBS and mounted with DAPI-containing medium (Vector Laboratories, Burlingame, CA, USA). Fluorescent images were captured using a fluorescence microscope (BZ-9000, Keyence, Osaka, Japan).

### *Ex vivo* autoradiography and TSPO distribution

Control rats and rats treated with CCl_4_ for 6 weeks were injected intravenously with [^18^F]FEDAC and then sacrificed after 1 h. Liver lobules were quickly removed and frozen with dry-ice powder. Frozen liver sections (10-μm-thick) were prepared using a microtome and thaw-mounted on glass slides. The sections were exposed to the imaging plate (BAS-MS2025; FUJIFILM, Tokyo) for 1 h. Radioactivity in the liver sections was detected by scanning the imaging plate using a BAS-5000 system (FUJIFILM). The liver sections after autoradiography were used for immunohistochemical staining with TSPO antibody.

The sections were incubated with a rabbit anti-mouse TSPO primary antibody overnight at 4 °C. After washing with PBS, the sections were then incubated with biotin-conjugated goat anti-rabbit IgG secondary antibody (1:1000; Invitrogen) for 1 h at room temperature, followed by tyramide signal amplification using a Fluorescein System (PerkinElmer). Fluorescence in the liver sections was detected using a FLA-5100 Fluoro-Imageanalyzer system (FUJIFILM).

### RNA preparation and qRT-PCR

Total RNA was extracted from frozen livers using Sepasol-RNA 1 Super (Nacalai Tesque, Kyoto, Japan) according to the manufacturer’s protocol. The quality of total RNA was checked by measuring the ratio of absorbance at 260/280 nm with a NanoDrop ND-1000 (LMS, Tokyo). The RNA then was reverse-transcribed into cDNA using PrimeScript^“^ RT reagent Kit (Takara, Shiga, Japan) according to the manufacturer’s instructions. cDNA were used as template for qRT-PCR using the Premix Ex Taq^TM^ (Takara) for the TaqMan method. qRT-PCR was performed on an Applied Biosystems StepOne system (Carlsbad, CA, USA). Target-specific primers and probes for signal transducers and activators of TSPO, TGF-β1, PDGF-β, TNF-α, and 18S ribosomal RNA (18S rRNA) were purchased from Applied Biosystems. The normalised Ct value of each gene was obtained by subtracting the Ct value of 18S rRNA. The fold change of each gene versus the control was calculated.

### Statistics

Data are expressed as the mean ± SE. Comparisons were made using one-way analysis of variance followed by Dunnett’s or Tukey’s tests. The analysis was performed using GraphPad Prism 5 software (GraphPad Software, La Jolla, CA, USA). Differences between groups were considered significant when the *p*-value was less than 0.05.

## Additional Information

**How to cite this article**: Hatori, A. *et al.* Utility of Translocator Protein (18 kDa) as a Molecular Imaging Biomarker to Monitor the Progression of Liver Fibrosis. *Sci. Rep.* **5**, 17327; doi: 10.1038/srep17327 (2015).

## Figures and Tables

**Table 1:** Assessment of fibrosis in livers treated with CCl_4_.

	n	Fibrosis score							Percentage of fibrotic regions (%)*
		0	1	2	3	4	5	6	
Control	4	4							0.36 ± 0.08
CCl_4_, 2 weeks	4			4					2.68 ± 0.16
CCl_4_, 4 weeks	5				4	1			5.82 ± 0.84#
CCl_4_, 6 weeks	4					2	2		12.45 ± 1.16#,##,###
CCl_4_, 8 weeks	5						2	3	13.79 ± 1.99#, ##, ###

^*^Data are represented as the mean ± SE.

^#^Significantly different (p < 0.05) from control.

^##^Significantly different (p < 0.05) from 2 weeks CCl_4_ treatment.

^###^Significantly different (p < 0.05) from 4 weeks CCl_4_ treatment.
